# Principles and Practice of Surgery

**Published:** 1848-06

**Authors:** 


### REVIEWS.

---

Art. IV.—*Principles and Practice of Surgery.* By the late Geo. M'Clellan, M.D. Edited by his son, John H. B. M'Clellan, M.D.; Philadelphia. Grigg, Elliott, & Co., 1848: pp. 432, 8vo.

The subjects treated of in this volume are the following: *the immediate effects of injuries upon the system*, *constitutional irritation*, *the effects of injuries upon the blood-vessels*, *erysipelas*, *furunculus*, *anthrax*, *abscesses*, *ulcers*, *burns and scalds*, *the effects of cold*, *wounds*, *tetanus*, *hydrophobia*, *poisoned wounds*, *syphilis*, *morbid growths*, *non-malignant tumors*, *malignant growths in general.* All of these subjects being comprised in the space of four hundred and thirty pages, it is evident that some of them are treated with a brevity by no means commensurate with their importance.

Under the first head are discussed the *immediate* effects of injuries on the nervous system in the form of *shock*, which may be confined to the parts injured, and the parts beyond in a peripheral direction; or may be felt by the nervous system of the whole body, and vary from slight disturbance to protracted and fatal collapse of the vital forces. The most dangerous form of shock, save that which overwhelms the individual at once, is termed by the author *insidious;* not more than one patient in a hundred recovers from it.

"A great joint has been torn open, a terrible compound fracture is present, or several of the large muscles and nerves have been lacerated, and yet no corresponding disturbance is manifested. The skin continues cool and transpiring; the tongue clean; and the secretions natural. Such cases may be compared to those which occur in the outbreak of yellow fever and other epidemics. Patients will be seen walking about, and perhaps attending to their regular duties, until the very moment when they drop down and die. During the epidemic typhus, or spotted fever of New England, the people fell down at the plough-tail, at the spinning-wheel, and the grist-mill. In one of our earlier yellow fever ravages, Dr. Rush met a friend walking the streets, with death depicted on his countenance, and had only time to accompany him home, and see him expire. During the cholera of 1832, a carpenter, who was carrying his tools to a workshop, was caught up by the police officers, because they saw the livid hue of his asphyxiated face, and dragged struggling to the nearest nospital, where he died, protesting that he would go back to his work.

"These may well be denominated cases of *insidious* shock. The symptoms are deceitful enough. Perhaps from the very suddenness and intensity of invasion, the springs of nervous energy are cut off—the foundations of life are undermined, and no further elaboration of the great principle of innervation can be effected. The actions of the system are allowed to go with apparant tranquility, until they wear down or exhaust all the power that has been stored up, and the forces sink into annihilation.

"The only symptom in addition to the above detail of circumstances upon which an intelligent surgeon can depend in the diagnosis of this kind of shock, is a semeiological one. The countenance of the patient is altogether unnatural. It presents an inquiring, anxious look about the forehead, eyes, and upper portions of the face, while all about the mouth and lips is smiling and composed. It resembles the mixed sad and cheerful expression which our great novelist, Dr. Bird, has attributed to Montezuma in his captivity, and by which some authors have attempted to characterize the whole of our aboriginal race—a melancholy smile about their lips, indicative of their fatal proclivity to destruction. There is another circumstance about the expression of the countenance, which will strike an intelligent observer. When you first approach the bed-side, the patient will look at you with a stare of alarm and suspicion; and he will repeat this expression constantly as often as you approach him to make any inquiry. No matter how long you remain in the room, whenever you attract his attention, his forehead will be drawn up, and his brows contracted into a scowl of suspicion, mingled with anxiety."

A more remote effect of injuries is *irritation*, which comes after reaction from shock, and even where no shock has been experienced. The hurtful impression is transmitted to the cerebro-spinal axis, and thence is reflected over all the functions of the body, constituting *general* or *constitutional irritation;* or through the same reflex action of the nerves, the results of this hurtful impression may be exhibited in some one organ more than another—as in the brain, by delirium, morbid vigilance, nervous apprehension; in the urinary organs, by paralysis of the bladder or suppression of urine; in the stomach, by nausea, and vomiting especially of undigested matters; in the liver, by arrest or perversion of secretion; in the heart, by palpitation, pain, great quickness of pulse; in the nervous system, by spasm and pain.

In the next section, on the effects of injuries on the blood-vessels, the author treats of inflammation. Here, as indeed everywhere else in the book, John Hunter is appealed to as the authority *sine qua non.* Dr. M'Clellan thinks that we have made no advance since Hunter's time; the microscopic anatomy of the present day, although he refers to it on all occasions, is considered by him as mere folly.

The best section under this head is that on senile mortification. The description of it, as it occurs in old gouty subjects, with ossification of the arteries, is as follows:

"A sensation of numbness or torpor, accompanied by an occasional prickling or neuralgia pains, and cramps of the muscles above, usually precedes the development of dry sphacelus, in the last extremities of one of the members, and symptoms of constitutional disturbance are also in most instances complained of. The patient has not been able to sleep quietly for some days; he has been anxious and restless, with loss of appetite and a sense of distress at the epigastrium. Chills or shiverings sometimes supervene, and a painful sensation of coldness and debility is complained of in the extremities. If the sphacelation appears before reaction, as it sometimes does, it is apt to make considerable progress before the patient or surgeon is made aware of it. Instead of a small black spot, as authors describe its beginning, a whole toe, and sometimes two or more will be found dead and shrivelled. This form is the real dry gangrene, and it may progress to a considerable extent before any degree of reaction occurs. But in the majority of cases an erysipelatous, or gouty redness, attended with a great deal of pain and sensation of burning occurs in the part before it mortifies. Indeed vesication occurs previously, and often the whole foot swells as in genuine gout. This is especially apt to be the case when the disease is excited by an injury, as from cutting the nails or corns, or wearing tight shoes, or exposing the part to sudden vicissitudes of temperature. The gangrene in these cases is sure to be humid, except at the very points or last phalanges of the toes, which may shrivel and become hard. The nails, however, speedily fall off, and leave the parts below and above moist and putrid. Most cases of this disease are excessively painful, especially when the gouty or inflammatory kind of reaction accompanies it. Although the torpid and uninflamed cases present nothing but a prickling numbness and slight neuralgic feelings which anodynes and stimulants can readily allay; those arising from, or complicated with, irritative inflammation of the parts above generally excite more distressing and excrutiating pains than any other disease in nature. The effects of large doses of opium, when prescribed by the celebrated Pott in such cases, were so admirable in the way of overcoming pain, and checking the progress of the disease, that it was denominated all over England, at one period, Pott's gangrene. Before the publication of Pott's treatise on this subject, bark and wine, and the most nutritious articles of food had been universally resorted to, for the cure of senile gangrene. He revolutionized the practice in a great measure, and ever since large and frequently repeated doses of opium with farinaceous food, and no stimulation, have been chiefly depended upon."

The cases accompanied with little or no irritation, will not be so amenable to Pott's treatment. In these, mild stimulants, nutritious food, and tonics, with warm and stimulating applications locally, succeed best. In the severer cases when opium is required, the author thinks it acts most beneficially when given in dry hard pills of solid opium. Not being readily dissolved when thus given, "it passes down below the stomach and applies itself to the nervous extremities over the whole mucous membrane of the bowels, and thus produces a composing effect upon the solids, with an inferior degree of narcotism exerted upon the brain and circulation." The truth is, we suppose, that when thus given, it is more stimulant than narcotic; the slow solubility of the "dry hard pill," rendering it equivalent to smaller doses of other forms, supporting the vital actions, instead of suspending them in narcotism. But opium or its substitutes, hyosciamus, stramonium, camphor, &c., are not the only remedies.

"Large doses of turpentine, of naphtha, or of creosote will often relieve miraculously after every other internal remedy has failed. By affording the requisite degree of diffusible stimulation to the nervous system, and rousing up the energies of the enfeebled capillaries, they appear to answer every indication in certain cases, which ought to be characterized for the benefit of practitioners. The cases to which allusion is here now made, all present the peculiar phenomenon of the patient holding the affected member in a depending position. No matter how bad and inflamed the parts may be, the limb will be held downwards, and frequently it will be impossible to prevent the sufferer from exposing it to the full blaze of a fire. It would appear as if the obstructed state of the arteries leading to the affected member, or their impaired condition of vitality prevented the free access of blood to the limb, and, therefore, required the aid of gravity and of warmth to promote the necessary access of blood into the capillaries. The minuter vessels and their controlling nerves being called on to make increased efforts to pump, as it were, the circulation through enfeebled arteries, necessarily generate the accompanying pain and irritative inflammation. The increased pressure of the column of blood from above, under the force of gravity, relieves them from the necessity of making such extraordinary efforts, comforts the irritated and exhausted nerves, and explains the advantages of a dependent position of the limb. The influence of warmth in some cases, and of internal carminative and diffusible stimuli, can also be satisfactorily explained on the same principle."

By and by the warm emollient applications, that seemed thus to appease irritation and promote the access of blood have to be laid aside; they favor tumefaction and infiltration, and the mortification advances rapidly under their use. But at the same time the dependent position of the limb becomes hurtful, and the patient must be placed on a sofa, or in bed, with the feet elevated. Cooling and astringent applications will now afford comfort; and benefit will be derived from bandaging, the skin being previously blackened by the nitrate of silver. When the gangrene is arrested, amputation may be completed, through the line of separation where nature has begun it; but, according to the author, never in the soft parts above. The successful cases of the latter sort which have been reported, he thinks have been mistaken. In illustration of the phenomena and treatment, of this disease, he subjoins notes of several cases: we quote the following:

"Some years ago I was called to see the late R—— C——, Esq., near Middletown, in Delaware. He was an old gentleman, about sixty-five years old, who had been a large farmer and hearty liver. I found him sitting before a hot fire on a low bed, leaning on his knees and holding on to both his feet, which rested on the floor. He was howling and shrieking with pains which no anodyne or topical remedy could relieve. It was impossible to keep him on the bed for a moment, and it had been so for nearly three weeks since his first attack. On examining his feet I found them excessively irritable and flushed over the insteps from the toes to the ankle. One of the toes on one of the feet, and two on the other, had become black and dry at the tips, and dark vesications had begun to form around their roots. His tongue was dry and furred, his skin hot, and his stomach oppressed and nauseated. His pulse could hardly be distinguished at the wrist or the ankle, for his arteries there were partially ossified. His femoral arteries were ossified also; and I had to ascertain the condition of the circulating powers from the temporals and carotids. His pulse in that way distinguished, proved to be quick and irritable but not strong. I found his bowels had been very torpid for some weeks, and no ordinary cathartics had proved sufficient. The evacuations procured by medicines had all been watery or scanty and tarry. I, therefore, recommended a twenty grain dose of calomel, in combination with twelve grains of rhubarb, which was afterwards repeated, and followed by oil and turpentine. Large evacuations of consistent fæces followed, to the great relief of the febrile excitement. I then blackened the whole feet, toes, and ankles, by repeated applications of nitrate of silver in solution (twenty grains to the ounce), and enveloped the affected toes in basilicon mixed with creosote. The result was a great immediate relief of the pains, and a marked subsidence of the general irritation. Opiates afterwards composed him to a reasonable extent, so that he could sleep in a horizontal posture and enjoy a tolerable protraction of his existence. Although he continued to suffer the dependent position of his legs in the daytime, still, under composure from opium, he could lie in bed over night, and thus he dragged out his existence of a few months longer, until he died without amputation."

In the succeeding chapters, on erysipelas, furunculus, and anthrax, we do not observe anything new. Under the head of gangrenous erysipelas is described a low form of inflammation, leading rapidly to purulent infiltration, destruction of texture, and mortification, occurring especially in laborers, who drink deeply, and eat largely of coarse unhealthy food. In all the severe forms of erysipelas, the author recommends free and deep incisions, and speaks highly of steam or vapor baths. Furunculus is discussed in thirty-five lines; anthrax in less than thirty!

In the section on abscesses, speaking of diffusion or infiltration of pus, he says:

"As soon as the presence of matter is detected by the sense of fluctuation, and an erythematic puffiness of the skin, the incisions should be made, and whenever the diffusion is very great, as along the whole extent of the thigh or forearm, counter-openings should be made at the opposite ends, as well as in the centre of the collection. Soft and porous, or sleazy cotton bandages should then be bound around the whole limb, so as to make equable compression. Openings should then be made through the bandage directly over each incision, and soft poultices or moistened pieces of flat sponge applied over the orifices. The matter, as well as the detached portions of sloughs, will thus be forced out through the orifice, while the opposite sides of the cavity will be compressed together at the same time by the bandaging. In this way an immense saving of time and suffering will be effected in the closing of the abcess."

Collections of pale, watery fluid, are designated by the term *lymph abscesses.* These may be connected with a lymphatic gland or vessel, but are not necessarily so; nor do they always imply exudation of fibrinous fluid. The term is very loosely applied. The collection may occur in the thighs or arms of relaxed and leucophlegmatic subjects. The following is a case of the kind.

"One enormous distension of this case occurred more than two years ago, in a corpulent washerwoman in Carpenter street. She was a mulatto, of about 45 years of age, but nevertheless she was decidedly leucophlegmatic in her temperament. One of her thighs was prodigiously distended, from the knee to the groin, with a sub-facial and intermuscular fluctuating effusion. She was taken to one of the Dispensaries after I had seen her, and an exploring puncture made through the vastus externus muscle. A gush of lymphatic fluid explained the nature of the case at once, and convinced the pupils that no cerebriform fungus was present."

An unhealed puncture of a lymphatic vessel gives rise to a small collection of this sort. Sometimes the fistulous opening in the vessel is congenital. The following case occurred in a young lady of seventeen.

"A very small, and almost imperceptible orifice existed in the skin directly over the anterior margin of the sterno-mastoid, in the middle of the neck. A common bristle could hardly be inserted into it, and it resembled very closely, one of the empty cutaneous follicles. A very small drop of transparent lymph could almost always be seen over this orifice, and every few minutes it would trickle down like a tear. On rubbing the neck with a slight pressure, especially upwards, this discharge would be considably augmented; and I took advantage of this to collect a few drops in a teaspoon. The fluid underwent an imperfect or flocculent coagulation, as I have found always to be the case on repeating Magendie's observations upon the lymph when extracted from the vessels of living animals."

The application of a red hot wire, or a pointed piece of lunar caustic, will generally procure obliteration of the orifice.

In the section on *congestive abscesses* there are some instructive cases detailed, of collections of matter connected with organic disease of the viscera—especially with obstruction at the ileo-cœcal valve, or ulceration and perforation of the colon. These abscesses in some instances are formed in the retro-peritoneal cellular tissue, and open like lumbar abscesses about the loins, in the thigh, or travelling over the spine of the ilium, appear on its dorsum. In other cases the collection takes place in the cavity of the peritoneum, and is circumscribed by the effusion of fibrin; these the author calls "circumscribed cold abscesses of the peritoneum," and directs attention particularly to them. We give a few of the cases, which will be found interesting.

"My excellent friend, Dr. Samuel Tucker, called me in consultation, some years ago, to see the son of a hatter in second street, about seventeen years old, who, after being troubled with obstinate costiveness for a long time, and colicky pains in the left iliac and lumbar regions, finally exhibited a deep seated fluctuating tumor projecting out towards the back part of the same lumbar region. I detected an abscess there, and on making a deep incision just in front of the quadratus lumborum muscle, a large quantity of very fetid pus escaped. A large lumbricoidus worm afterwards escaped through the orifice, and the odor of fecal matter for a long time exhaled from it. But, finally, the abscess closed, and the bowels were no longer subject to derangement.

Dr. Charles Schwartz lately called me in to a similar case, which occurred in the person of a German tailor, in Race street. After having been ineffectually treated for eighteen days, by a homœopathist, for inveterate constipation and pain in the left lumbar region, which was mistaken for nephritic calculus, he sent for Dr. Schwartz, who at once detected a deep seated tumefaction in that region. He resorted to active antiphlogistics, cathartics, and injections, but could not prevent the tumor from suppurating, although he succeeded, to some extent, in evacuating the bowels. I opened the collection just in front of the anterior margin of the quadratus lumborum muscle, and evacuated a very thick offensive pus, after which the bowels resumed their natural functions, and all the pain subsided."

"In the year 1825, I was sent for, to introduce the catheter for a Mr. Thomas, in Filbert street, who was supposed to be laboring under a retention of urine. I found him with a large fluctuating tumor in the regio pubis precisely like an over distended bladder, and he complained of all the symptoms of a painful retention. I therefore introduced the catheter, as I thought fairly into the bladder, but not more than a tablespoonful of high colored urine escaped, and as the symptoms were not at all relieved therefrom, I introduced the left index up the rectum, where I found what seemed to be an enormous distention of the lower fundus of the bladder. On making percussion upon the swelling in the regio pubis, I could distinguish a palpable fluctuation, through the swelling felt in the rectum, and I could not doubt that the bladder was over distended. Still the catheter was in the bladder, and no more urine would escape. On withdrawing the catheter, it was found to be unobstructed by coagulated blood or any other substance. I afterwards introduced a larger one on my next visit, but the same result followed. The next day Dr. Physick visited the patient with me, and introduced a silver catheter with the same effect. He then carried his left index as high up against the lower fundus of the bladder as possible, and forced up the catheter so as to be certain of its full passage into the bladder. In doing this, he felt some barrier giving way before the point of the catheter, and the patient, at the same instant, cried out that he felt his bladder tearing. On withdrawing the catheter, blood for the first time appeared in its eyes and cavity, and we were persuaded it must have made a false passage. The ensuing day, the doctor repeated the same effort ineffectually with another catheter, and he, therefore, directed me to tap the vesical tumor above the pubes. When I had introduced a trocar, to our astonishment, a sero-purulent fluid escaped instead of urine, and the collection proved to be a large circumscribed abscess of the peritoneum, gravitating down into the cavity of the pelvis, and floating around the bladder, or rather the empty bladder floated within it. In a few hours the man died. He was a young man twenty-three years of age, the son of Mr. David Thomas, a very respectable citizen. We found, on careful inquiry, that he had swallowed a date stone about two years before, and ever after complained of feeling it lodged in his right groin. For about two weeks before I first saw him, he had been attended by Dr. Gillingham for colic and constipation, and after the application of a large blister, the swelling of the lower part of the abdomen had appeared and stranguary had supervened. Ineffectual efforts had been made to relieve the stranguary, by warm baths and opiates, and Dr. G. was obliged to leave town, when I was called in, on account of his own health. On examining the body, we found a large mass of coagulated lymph, partly organized around the appendix vermiformis, and the veritable date stone lodged within the cavity of the appendix and but slightly projecting into the cæcum. The exhalation of puriform matter had originated there and gravitated downwards. Surrounding barriers of adhesion had formed between the intestines and the lining of the abdominal walls in front, and the abscess was confined to the lower part of the umbilical, the hypogastric, and the pelvic regions. It was the lower part of this abscess which we had felt through the rectum, and mistaken for the distended lower fundus of the bladder. The free serous surfaces, looking into the abscess, were everywhere thickened and opaque, and incrusted with a scaly or broken false membrane of coagulated albumen or fibrin. The injury which the catheter had done to the bladder consisted in the forcing of its point into the orifice of the right ureter, where it had torn up the mucous membrane to a considerable extent and allowed some of the urine to infiltrae under it, so as to puff it up like a blister."

Dr. M'Clellan divides ulcers into nine varieties: the healthy, inflamed, irritable, indolent, varicose, scrofulous, phagedenic, sloughing, and specific. How long will our books on surgery continue these perplexing and unscientific divisions?

In the case of wounds about the hands and feet, he objects decidedly to the practice of cutting down upon the principal artery of the limb in order to arrest hemorrhage. In the whole course of his practice, he has met with but two cases where it was necessary to take up the radial artery, and four or five where it was necessary to take up the tibial arteries. Pressure, properly applied, or dilatation of the wound and ligation of the bleeding vessel, have always proved sufficient.

In connection with gun-shot wounds, several interesting cases are detailed.

"In Mr. Cameron's case in Lancaster, when he was shot apparently through the body by a musket ball, I was called out the same night to consult with my friend, Dr. Atlee. We found that the ball had traversed round the abdomen, across from the right to the left side, glancing under all the muscles, probably without laying open the peritoneum. The ball was extracted by Dr. Atlee just under the skin, where it had attempted to make an exit, and the whole track and last orifice healed without suppuration. The abdomen however became tumid and painful from the local shock or contusion, and required active depletion and purgatives for relief. The orifice of entrance was very small, and readily healed."

"In the case of Mr. Griswold, the U. S. mail agent, who fell under my care for three bullet wounds, received in the night, at Bristol, in 1835, the muzzle was held within a yard of his face, and one ball entered the alæ of the nose, and passed through the nostril into the substance of the second cervical vertebra, whence it was afterwards dislodged; the second passed into the orbit of the eye, on the same side, through the lower lid, just escaping the bony edge of the orbit, and passed back into the sphenoid bone, where it still remains; and the third struck the front surface of the upper jaw bone just below the orbit, and passed back into the tuber maxillare, where it still remains embedded. He fell, after one partial reaction, into a secondary stage of overwhelming shock, which persisted for some hours. He had no hemorrhage, except a little from his nose and throat, nor any pain or fever afterwards. The nerves of special and common sensibility were entirely paralyzed on that side, and have never since recovered any power. But the muscular expressions are all perfect, and the scars of two of the wounds, the first two, are hardly visible. They healed like incised wounds; but the third orifice being contused against the body of the upper jaw bone, sloughed at its edges, and left a deep scar."

A valuable lesson is taught in connection with the next case.

"Nothing can be more reprehensible than to disturb the dressings of a wound in which a hemorrhage has been arrested. As long as the parts are dry and quiet, let them alone until the subsequent discharges begin to loosen the dressings. By that time the processes of adhesion will have permanently closed the orifice in the wounded artery, and prevented all secondary hemorrhage. During the riots in Southwark, I dressed a horrid canon-shot wound in the right groin of a young soldier from Germantown. He had been struck there with a broken piece of cast iron, with the fragments of which one of the cannon in the hands of the mob had been loaded. The whole fore part of the fleshy substance of the groin, in contact with Poupart's ligament, had been torn away, and a prodigious rent made across the artery, with a hole in the vein. He had flooded the floor, on which he lay, with gore, and was deadly faint when I arrived. It was late at night, and the rioters were all around. I, therefore, applied graduated compresses, and bound them down with a spica bandage. But it required two hours constant nursing, with his head and shoulders in my lap below the bedside, and the incessant use of cordials and brandy, to revive him. During his reaction the poor fellow was delerious, and wanted to go home. He had no return of hemorrhage, however, and by morning I got him so far recovered, as to have him transported, in a carriage, to one of the hospitals. There, however, somebody stripped off his bandage and pulled away the compresses, to see how I had dressed the wound! The blood gushed out in a torrent, and the second fainting process carried the patient off to a better world."

The section on syphilis is the only one in which the author demurs to the authority of Hunter. On this subject he subscribes to the doctrines of Carmichael. He thinks with him that "there are four specifically distinct kinds of disease resulting from as many distinct poisons;" and further that his "nosological arrangment and nomenclature are consistent with the facts and explanatory of the errors of Hunter." Accordingly he treats of papular, pustular, phagedænic, and scaly or true syphilis. The distinct existence of the latter has been conclusively established by Ricord, and Dr. M'Clellan does not doubt "that others will finally be established on equally strong data."

"It is impossible form the very nature of things, that septic poisons should not vary. The elements and products of secretion vary in the different parts of the same complications of organs, even in the same sex; and when we take into consideration the commixture of these secretions, after irregular and promiscuous intercourse between the most filthy and negligent of both sexes, we can experience no difficulty in concluding, that the chemical or putrefactive reactions which take place among them, must be productive of diversities of animal poison. Each form of the virus on being generated, may continue to be propagated for an indefinite period by contagion: or it may finally wear out or be lost for want of means of communication; and then a new species be formed afresh from another concomitance of circumstances. In the same way we may conceive of a number of contagions going on collaterally at the same time, through different members of the same community, although not mixing with each other respectively."

Touching specifics in this disease, he says:

"It may appear disheartening to some who have cultivated medicine on empirical ground, to learn that no antidote or real specific has ever yet been discovered for any form of syphilis. Everything that has ever done good has produced its curative effects on the same general principles which govern us in the management of other diseases. A well regulated diet, bloodletting, purgatives, diuretics, and especially diaphoretics and alteratives, have all been brought under contribution with complete success, in the different stages and forms of syphilis. The aborigines of this continent were proverbially successful in their treatment of its worst shapes, by the use of simple diaphoretic herbs and roots, aided by the vapor bath. In fact the so long celebrated decoction of the woods, as they are called was originally borrowed from the Indian practitioners near the earliest settlements on this continent. Alteratives, the class of remedies perhaps most extensively depended upon at present, produce their beneficial effects not merely by changing the actions of the solids, but also by influencing the secretions and exhalations so as effectually to eliminate the poison from the system. Mercury is only one of this class of remedies, and its great and peculiar advantage, as we shall see, consists in the fact, that it possesses extraordinary powers in dispersing chronic indurations and quickening the action of the capillaries. In all cases where there is a want of indurated or unorganized lymph, or where the capillaries are in an over excited state, the alterative impressions of mercury will prove positively injurious; and then a simple course of diaphoretics, aided by appropriate antiphlogistics or tonis, as the state of the system may require, will prove as decidedly curative as any searcher after specifics can desire."

Mercury is not merely inapplicable, but positively hurtful, in the papular, pustular, and phagedenic varieties, both in the primary and secondary forms. In all stages of the *scaly* syphilis, the true syphilis of Hunter and Ricord, it is "peculiarly appropriate, not because it possesses any antagonism to the infecting virus, but simply in consequence of the fact that the chronic and unirritated form of the symptoms indicate such a condition of the system, as is found appropriate to the favorable influence of mercury in all other diseases." In the true syphilitic bubo, a longer use of mercurials will be required than for simple chancre; and sometimes pressure, caustics, and blisters "will be necessary to facilitate the action of mercury upon the indurated base and edges of the consecutive ulcer." Dr. M'Clellan is not very partial to the proto-iodide of mercury; he prefers the old favorites of the profession.

Of the treatment of the *phagedenic* syphilis the author says:

"Since the publication of the earlier editions of Mr. Carmichael's work, a very great improvement has been made in the treatment of the primary symptoms of phagedena. Instead of the temporizing treatment recommended by him of blood-letting, fomentations, poultices, antimonials, purgatives and opiates, we now proceed immediately to destroy the whole morbid surface, by potential cauteries. A bridge of basilicon ointment is smeared around the edges, and over the surface of the neighboring skin and then lint dipped in pure nitric acid is applied firmly over the sloughing or corroding surface. Emollient poultices afterwards speedily detach the sloughs and present a suppurating and granulating surface. In short, this efficient plan of treatment at once converts the diseased into a perfectly healthy ulcer, and protects the constitution from all contamination in most cases. The secondary eruptions being accompanied with much febrile reaction, always require depletion at first, and generally, in the form of blood-letting and purgatives. Antimonials and diluents afterwards come in as adjuvants, and finally, sarsaparilla with Dover's powder, or other sudorifics. The ulcers on the skin should be cauterized with caustic potass at their morbid edges and surfaces from time to time, and emollient poultices of slippery elm applied until the surface becomes healthy. Afterwards soap cerate, or Turner's cerate, spread on lint or linen will speedily effect cicatrization."

According to the author this disease was very common in Philadelphia in 1818–19 and 1820, and again in 1827–8, but has since been less frequently met with. He details several cases that occurred in his private practice. The first of these he treated in 1829 in the person of an Irish school-master named Kelly, a young man of temperate habits and of sound constitution. This case first led him to regard favorably the doctrine of the distinct origin of the disease. The other cases are thus detailed:

"At the White Bear Inn, in market street, within half a square of Kelly's schoolroom, and where I know the poor fellow had been in the habit of eating his dinner in ordinary, or, at least, occasionally, the then proprietor of the house, Mr. Caldwell, first sent for me to see a black and painful spot on his prepuce, which had broken open, or, as he said, burst the night before and discharged a bloody sanies and a very offensive odor. I immediately discovered the first stage of sloughing phagedena, and having shortly before seen Mr. Welbank's paper in the Medico-Chir. Transactions, I tried his plan of treatment by applying the concentrated nitric acid. This succeeded like a charm. The moment the slough which it caused came off under a flaxseed poultice, a crop of healthy granulations shot out and repaired the breach. This succeeded in preventing all secondary disease, as it has done in every other case in which I have seen it tried as early after the exposure. But as soon as Mr. Caldwell had got well, one of his boarders, Mr. G. W. J., a wholesale shoe dealer in market street, took his bed and sent for me. He was a perfectly healthy and respectable young man, of twenty-six years of age, accustomed to no kind of dissipation. About a week after visiting the same chambermaid, who had certianly infected the landlord, and probably the poor schoolmaster, he began to experience a distressing pain in the inner side of his right thigh just below the groin, in which region he soon detected a black speck or tubercle. This rapidly enlarged, cracked open., and discharged an offensive bloody matter. He had consulted the nearest apothecary, who gave him some balsamic mixture to apply on patent lint, but before I saw him the slough had extended to the size of half of one of his palms. I lost no time in applying a piece of patent lint, cut to the size of the whole surface, and dipped in pure nitric acid. A surrounding bridge of basilicon ointment protected the sound skin, and a poultice afterwards detached an enormous slough from a subjacent healthy ulcer. After this ulcer had healed without the development of buboes, I was confident that he would escape secondary symptoms; but unhappily, in about three months he came to me with fever, and large wheals or tubercles on his forehead, face, breast, and shoulders, which scabbed over like rupia, and were followed in some points, by enormous phagedenic ulcers. His throat and nostrils ulcerated, the spongy bones exfoliated, his joints become painful, the whole category of symptoms laid down by Carmichael supervened. I had consultation on consultation with my professional friends, sent him to the sulphur springs, the sea-shore, and the country. But he finally died, after three years suffering, with marasmus and perichondrial swellings about the larynx, without any injurious course of mercury ever having been instituted. A few carefully conducted trials of an alterative course, at the suggestion of some of my consulting friends, failed in affording any more than plliative relief even in the declining stage of the symptoms."

We have seen two cases of this disease: one was in a youth of fifteen; the other, in a very robust healthy looking man of twenty-five. The latter had lost the prepuce, and about two-thirds of the glans penis, when we saw him; he recovered, under the application of nitric acid and poultices, without further mutilation of the organ; but what is his subsequent history we do not know. There was nothing in the habits or general health of either of these patients to account for the character of the disease. It could only be referred to something peculiar in the virus.

Dr. M'Clellan does not agree with Ricord and his followers, that mercury is inapplicable and iodine exclusively beneficial in *tertiary* syphilis, or "in the secondary symptoms of the second order of parts," as Hunter phrases it. Iodine is a good adjuvant of mercury, "but no practitioner has ever yet found it possible to dispense with the truly heroic remedy in the last and most protracted form of syphilis. Even in the last or declining stages of the other forms of venereal diseases, for which mercurials are universally considered inappropriate, the same combinations often come in as successful remedies." Iodine and sarsaparilla seem, he says, to have the power of depriving mercury of its most injurious properties; but iodine can only be substituted for mercury in those irregular and anomalous affections thought by Hunter to be allied to scrofula. Arsenic, in some instances, has done more good than either iodine or mercury.

The proportion of cases in which secondary symptoms occur after cure of indurated chancre by mercury is, according to Ricord, eighty-six in a hundred. Dr. M'Clellan thinks that in this country the proportion is about fifty or sixty in a hundred; and he asks if the difference is not owing to Ricord's constant practice of inoculation. He also brings forward some instances to show that the remote or tertiary symptoms of Ricord may be transmitted from the father to the children without intermediately affecting the mother.

The last subject treated of in this volume is that of *tumors*, the discussion of which occupies more than a hundred pages. The author makes two divisions, non-malignant and malignant. The first includes encysted, cystoid, sarcomatous (embracing adipose sarcoma and fibrous tumors,) cartilaginous, and osseous tumors (exostosis, spina ventosa, and osteo-sarcoma); the second includes scrofula, scirrhus or carcinoma, medullary cancer or medullary fungus, and melanosis or black cancer.

This is not offered as a complete or scientific arrangement, but one which subserves the purposes of discussion. A good many notes, containing accounts of some of the author's operations, are given in this part of the volume, and we shall confine ourselves mainly to these. Under the head of encysted tumors, we have the following account of an operation for *hydrocele of the neck.*

"A most interesting case of the so-called *hydrocele of the neck* presented itself to the author, about four years ago, in the person of Mr. T.—, of Kentucky. The disease had commenced many years before, and during the last two preceding his arrival here, had increased so rapidly as to cause considerdeformity, and to interfere somewhat with respiration and deglutition. The tumor gave the appearance of a uniform œdematous-like ênlargement of all the parts adjoining the thyroid body. The skin pitted under pressure, and an indistinct feeling of fluctuation was communicated to the fingers of each hand when pressed upon the opposite sides of the enlarged space. It was pronounced to be a cyst, containing a watery fluid: and an exploratory puncture was made by introducing a a narrow bistoury ovei the edge of the sterno mästoid muscle. At least a pint of a thick greenish serous fluid, tinged with blood, escaped; and an injection into the sac of warm water brought away a number of albuminous flakes through the aperture which was then closed. Two days after the operation, the wound had united, but the sac had filled up to its former size, and seemed to be much inflamed, whilst the difficulty of breathing had increased. During the whole of the succeeding week, he had much pain and uneasiness in the part, and suffered from severe, and even alarming, constitutional irritation. At the end of that time, however, more distinct fluctuation being perceived on the side opposite to the former puncture, a free incision let out a quantity of bloody serum, to the great and immediate relief of the dangerous symptoms. From this time, he continued to improve rapidly, and free suppuration throughout the sac resulted in its complete obliteration.

"He returned to Philadelphia within the last year, and, upon examinotion, the parts were found to be still somewhat enlarged and indurated—but there was no return of the fluid collection. Frictions of iodine ointment, with repeated leechings, and a long course of alteratives, almost entirely removed this induration, which was nothing more than the remains of the closed sac, and restored his neck to nearly its natural shape."

Dr. M'Clellan objects to the use of the seton in these tumors, as producing excessive inflammation, and says he has succeeded better by making free incisions and then applying nitrate of silver to the inner face of the cyst. Theoretically, the seton is more manageable than incision and cauterization, and we are much inclined to think it would be so practically. We have seen it successfully used in one case.

In connection with cholesteatoma we have this remarkable case:

"Some years ago, I was called down to Milford, in Delaware, to visit the late Col. P——, then confined to his bed with a singular complication of diseases. He had strictures in the urethra and rectum, stone in the bladder, with diseased prostrate, and incipient cataracts in his eyes, besides two large malignant and ulcerated lupuses on his cheeks. But the worst of all his afflictions proceeded from a pendulous white or pearly-looking tumor, about two inches long, which hung down from the posterior half arch of his soft palate on the left side, and fell upon the top of the larynx. This kept up a constant sense of suffocation and difficulty of deglutition. The poor old gentleman could never repose, not even for an instant, in the recumbent posture. He had to be propped up on pillows, with the left side of his head and neck downwards, so as to prevent the tumor from falling by gravity into the larynx. I excised the tumor with immediate relief to his most troublesome and dangerous symptoms, by means of a hook and blunt-pointed bistoury, It had a narrow pedicle, and was pyriform in shape, resembling, very closely, a single large pearl glass pendule of the ear-ring sometimes worn by females in vulgar life. It was entirely destitute of vessels, and covered with a very thin transparent membrane, The firm and lamellated cholesterine of which it was composed, looked precisely like opal or iridescent pearl, as it hung within the throat. This was a genuine cholesteatoma. He had large indolent ulcers on his legs, both of which were also covered with a thick coat of shining white cholesterine, evidently of the same character as the contents of the pendulous sac in the throat."

Dr. M'Clellan has the credit of demonstrating fully the practicability of extirpating the parotid. He removed the entire gland in eleven cases; in one, the patient "sank under coma in the fourth day after the operation, from the effect of the ligature around the common carotid artery;" in another, the patient died three years aftewards from return of the disease. The subjects of the remaining cases are still living.

The editor has also extirpated this gland, for an immense tumor involving the pneumo-gastric nerve, which was divided without any untoward results.

We find the subjoined case in the section on cartilaginous tumors of the bones.

"One remarkable case occurred to me, about eight years ago, of an enchondroma in the interior of a flat bone. The Hon. Mr. Allen, of Cleaveland, Ohio, then a member of the U. S. Congress, brought his son to me, a fine lad, of about seven years of age, with a broad swelling in his left temple, which had been for eighteen months gradually enlarging, and attended with a troublesome determination of blood to the head. It had been pronounced an incipient exostosis of the skull by some distinguished practitioners in other cities, and the little fellow was brought to me to undergo an operation. On a careful examination, I found a gradual rising of the external table of the lower portion of the parietal bone over a large circular space, of about two and a half inches in diameter, with a prominent and elastic portion in the centre as large as a twenty-five cent piece. This was destitute of a covering from the external table of the skull, and felt like a fibro-cartilaginous tumor with three peculiar fixed and hard lumps in its substance, which I compared to round-headed nails standing np through the elastic mass from the firm internal table below. As the disease was then making progress, and threatened the integrity of the cerebral powers, I consented to the wishes of the parents, and undertook the operation. After dissecting away the angles of a triangular or T-like incision, and exploring the pericranium, I revealed the surface of the fibrous mass as it was exposed in the centre of the swelling, from which the entire substance of the external table had been removed by absorption. I then made a circular incision around the whole circumference of the tumor through the external table, with a small circular Hey's saw, and pryed away the bony crust included within this circle by an elevator. On dissecting up the flat and firm tumor which was then exposed from the internal table of the bone below, I had to saw off the three bony knots or studs I have alluded to, from the internal table. The whole tumor was then fully detached, and proved to be chiefly fibrous with cartilaginons deposits among all the interstices. The three knobs were eburneous or ivory-like exostoses, and the whole substance of the exposed tabula vitrea at the bottom of the wound proved to be composed of radiated or stellated fibres of eburneous and shining spicula. I therefore carefnlly cut through these by the gentle application of a small Hey's saw, and raised and completely detached them from the dura mater below by the forcéps and elevator. The main branch of the middle temporal or meningeal artery bled freely, and I was obliged to secure it with a fine ligature. The flaps were then reapplied, and contracted speedy adhesion to the dura mater, and the little fellow left town for his home in three weeks, with a soft pulsating space in the situation of the wound, but with no symptom of inconvenience. The disease has not returned, and I hear that so much ossific matter has been re-deposited below the scalp as to suppress the pulsations there completely."

The shining and ivory-like fibres mentioned in this narration, sometimes form under the surface of scirrhous and even fibrous tumors, and, according to the author, ought always to be removed.

Some of Dr. M'Clellan's most important operations were for excision of the ribs, and the removal of the whole or a part of the upper and lower jaws. In the latter cases, he has never found it necessary to tie the carotid as a preliminary step. We hold that it would be mal-practice in most of such cases; the only good it can do, is to enable a surgeon to say that he has tied the carotid a certain number of times—a most pitiful piece of ostentation.

We quote the author's remarks on the general treatment of cancer.

"Although the constitutional treatment of carcinoma usually resorted to by the profession, consists chiefly of alteratives and tonics, a considerable variation from this course is frequently required in particular cases. In the earlier stages, before the peculiar cachexia has been manifested, it is always best to alleviate the irritation by a well regulated diet, combined with laxatives and occasional diaphoretics. As long as there is any degree of plethora, or vascular excitement present, it will be best to confine our patients to vegetables, farinaceous food, and milk diluted with water. Great attention to cleanliness, and warm clothing, should be observed. The flannels should be changed and aired every night and morning, and ablutions with warm water should be practised every morning before putting on the dress. Often great benefit will be derived from a general sponging with warm water acidulated with nitro-muriatic acid followed by frictions before retiring to bed at night. By such means the cutaneous transpiration will be constantly maintained in a state of healthy vigor, which is most essential to the counteraction of all malignant growths. After the constitution has begun to flag, and the symptoms of cachexia have supervened, the use of light and digestible animal food will be required in combination with the farinacea, and the vegetable tonics. If any tendency to anemia, hower, be manifested, chalybeates will become the best invigorators. The carbonate of iron in full doses, combined with small proportions of rhubarb and ginger, or aloes, will in general prove the best formulæ."

Locally "the application of leeches from time to time to allay vascular engorgement and irritation, the use of cooling and astringent lotions at first, and of mild sedative plasters afterwards," constitute the palliative treatment. By combined local and general means much may be done to check the progress of these formations; and it is in the chronic not the acute stage that the knife should be used.

With these extracts we leave the reader to form his own judgment concerning this volume. We have purposely abstained, save in one or two instances, from anything like criticism either of the matter or the manner. Still we must say that we do not regard it, like one of our contemporaries, "as an original, philosophical treatise on surgery," or as evincing any very "profound learning" on the part of the writer. It is to be regarded—and we beg the reader to bear this in mind—not so much as a grave treatise as a reproduction of the author's extemporaneous lectures, delivered some years ago in one of the Philadelphia Colleges. Its style is that of the lecture room rather than the closet; and its faults, we would fain believe, are those of the lecture room rather than the closet. Besides the intrinsic evidences of this, we have the direct avowal of the author that in its composition "he was obliged to shut himself up for an hour or two at a time, and reproduce his former train of ideas and facts as he was in the habit of giving them extemporaneously in the lecture room." It is then this "former train of ideas and facts" with the changes wrought by lapse of years; it is his lectures as they would be now if they were now to be delivered—a reproduction of them, with a slight (and in some cases very slight) incrustation of later knowledge, gleaned from later reading and experience. As such, then, the reader must recollect that these pages lack the speaker's look and tone, his appropriate attitude, his impressive gesture, his rapid and impetuous flow of language—in a word, that, with much of the *abandon* of the lecture room, they lack all the nameless attributes of the living lecturer, and all the nameless attractions of an attentive audience. C.

